# Importance of Pre-Transplant Colonoscopy in Renal Transplant Recipients

**DOI:** 10.14740/jocmr1934w

**Published:** 2014-09-09

**Authors:** Amelie Therrien, Jeanne-Marie Giard, Marie-Josee Hebert, Mickael Bouin

**Affiliations:** a Department of Medicine, Division of Gastroenterology, Centre de Recherche du Centre Hospitalier de l’Universite de Montreal (CRCHUM), Montreal, Canada; b Department of Medicine, Nephrology - Transplantation Unit, Centre de Recherche du Centre Hospitalier de l’Universite de Montreal (CRCHUM), Montreal, Canada

**Keywords:** Colonic polyps, Colonoscopy, Colorectal neoplasms, Kidney transplantation, Screening

## Abstract

**Background:**

Current recommendations for colorectal cancer screening for kidney transplant candidates are the same as for the general population. However, few studies have established the prevalence and characteristics of colorectal polyps in this population. The aim of this study is to describe the prevalence and characteristics of colonic lesions detected by pre-transplant colonoscopies in our kidney transplant population.

**Methods:**

A retrospective study was conducted from January 2007 to December 2009 at the Centre Hospitalier de l’Universite de Montreal (Canada). Inclusion criteria are all renal transplant recipients with a test for colorectal cancer screening in the 5 years preceding the transplantation. Patients benefiting of a second transplantation were excluded. The files were reviewed for clinical data, including colonoscopy indication, endoscopic and pathologic results. Advanced lesions were defined as adenomas of 10 mm or greater or with a villous component. Polyps were considered proximal if they were at the level of or above the splenic angle.

**Results:**

This study includes 159 patients. A pre-transplant colonoscopy was performed in 40% (n = 64). Polyps were present in 32.8% (n = 21) of colonoscopies and 66.7% of them showed adenomas. Advanced lesions were present in 6.25% of the exams. Finally, 66.7% of patients with polyps had at least one proximal lesion.

**Conclusions:**

The prevalence of colorectal polyps before transplant is high among renal transplant recipients. The high prevalence of proximal lesions supports the need for total colonoscopy.

## Introduction

End-stage renal disease (ESRD) affects an increasing number of people in North America [1] and can be associated with an increased risk of cancer [2-5].

Colorectal cancer remains the third most prevalent cancer in North America [6]. Whether the incidence of colorectal cancer in the ESRD population is similar [3-5] or higher [7-9] than in the general population remains to be established. Currently, screening recommendations for kidney transplant candidates are the same as those for the general population [10, 11]. However, there is an association between the severity of renal failure and the global risk of neoplasia [3]. A recent study has shown an association between ESRD and increased prevalence and severity of colorectal lesions found in patients with positive fecal occult blood tests (FOBTs) [12]. Several variables may influence the incidence of colorectal lesions in kidney transplant candidates, especially diabetes [13], obesity [14], statin use [15], immunosuppressive drugs [16, 17], etiology of primary kidney disease [4, 5] and type and length of dialysis [4, 18].

The finding of a colorectal neoplasia excludes patients from the transplant list for a period of 5 years from clinical remission [11]. However, adenomatous polyps are not a contraindication to transplantation and their prevalence among patients awaiting kidney transplantation is unknown. To our knowledge, only one study has looked into this matter; it did not show a statistically significant increased risk of colorectal polyps in this population, but the small number (n = 57) does not allow us to draw definitive conclusions [19]. Moreover, growing evidence is pointing to an increased risk of colorectal neoplasia after transplantation [4, 17, 20-22]. Due to the well-described natural history of adenomatous polyps, it appears necessary to evaluate the prevalence of polyps before kidney transplant to better adjust polyp surveillance post-transplantation.

Our goal was to describe the prevalence and endoscopic and pathologic characteristics of colonic lesions detected by pre-transplant colonoscopies in our kidney transplant population. Sub-analyses were completed to determine the differences between patients with and without colonic polyps.

## Materials and Methods

We performed a real-life observational study of all kidney transplant recipients transplanted between January 1, 2007 and December 31, 2009, at the Centre Hospitalier de l’Universite de Montreal (CHUM, Montreal, Canada). Our center’s ethics committee approved the study on July 1, 2011.

### Patients

All consecutive patients who received a kidney transplant within the study period were eligible. On a yearly basis, approximately 70 renal transplants are performed at our center. We planned to study about 200 patients. Patients who had already received a kidney transplant were excluded. The list of patients with a pre-transplant colonoscopy was obtained from the electronic clinical database of the CHUM renal transplant department.

### Data collection

For each patient, the following data were collected from the electronic clinical database and the patient’s medical file: 1) clinical characteristics; 2) colonoscopy reports done less than 5 years before the transplant. We reviewed each patient’s medical file looking for the following comorbidities: BMI, diabetes, systolic left ventricular dysfunction (< 50%), statin use, levothyroxine use, alcohol use (current vs. occasional vs. previous), smoking status (active vs. previous vs. life-long non-smoker), family history of polyps or colorectal cancer, personal history of inflammatory bowel diseases, polyps, colonic surgery, cholecystectomy, colorectal cancer or another neoplasia. Finally, the etiology of the kidney failure, the type and length of dialysis treatment and immunosuppressive drug use were also reviewed.

We collected patients’ age at the time of the colonoscopy, indication of the exam (screening vs. diagnostic exam), colon segments visualized, and endoscopic and pathologic results. We have considered the exam as a pre-transplant screening if clearly indicated on the report and if there was no mention of gastrointestinal symptoms (diarrhea, change in habits stools, hematochezia, melena, constipation) or anemia as indication. We also searched for other screening tests (FOBT, sigmoidoscopy, double-contrast barium enema (DCBE)). However, FOBT and sigmoidoscopy are not commonly used in Quebec for colorectal cancer screening. We collected the results of DCBEs and any subsequent colonoscopy. Advanced polyps were defined as adenomas of 10 mm or greater or with a villous or high-grade dysplasia component. Polyps were considered proximal if they were at the level of or above the splenic angle.

### Analysis

We compared the characteristics of groups with and without colonoscopy. In the group with colonoscopy, we compared the clinical characteristics of subgroups with and without polyps. We also compared patients according to their age (> 50 years old vs. < 50 years old) and the indication of colonoscopy (screening vs. diagnostic). Missing data were considered missing at random. There was no missing data for the variables of interest presented in Tables 1-4, except for pathology for which a distinct group was created (“unknown pathology”). For the clinical characteristics not shown in the tables, missing data ranged from 0.6% to 5% of the sample. Statistical analyses for continuous variables were means with standard deviation (SD) and confidence intervals (CIs) of 95%; we used *t* tests to compare them. The Chi-square test was used to compare categorical variables. A P value of 0.05 was considered statistically significant.

**Table 1:** Characteristics of Patients and Comparison of Groups With and Without a Colonoscopy 5 Years or Less Before Transplant

	Global population % (n = 159)	Colonoscopy % (n = 64)	No colonoscopy % (n = 95)	P value
Male	64.2 (102)	75 (48)	56.8 (54)	< 0.05
Age at transplant (years), mean and standard deviation	48.1 (SD 12.5)	55.6 (SD 8.7)	43.1 (SD 12.1)	< 0.05
≥ 50 years old	48.4 (77)	70.3 (45)	33.7 (32)	< 0.01
Body mass index (kg/m^2^), mean and standard deviation	26.3 (SD 5.5)	28.3 (5.3)	24.9 (5.3)	< 0.01
Type II diabetes	15.7 (25)	29.7 (19)	6.3 (6)	< 0.01
Diabetic nephropathy	13.2 (21)	23.4 (15)	6.3 (6)	< 0.05
Dyslipidemia treated with statins	49.1 (78)	60.9 (39)	41.1 (39)	< 0.05
Dialysis	88.7 (141)	90.6 (58)	87.4 (83)	0.70
Immunosuppressive use	14.5 (23)	17.2 (11)	12.6 (12)	0.57

P value: comparison between “colonoscopy” and “no colonoscopy”.

**Table 2:** Comparison Between Patients With and Without Polyps

	Polyps n = 21 (%)	No polyps n = 43 (%)	P value
Male	76.2 (16)	74.4 (32)	0.88
Age at transplant (years), mean and standard deviation	57.3 (SD 6.5)	54.8 (SD 9.5)	0.21
≥ 50 years old	76.2 (16)	76.7 (33)	0.96
Body mass index (kg/m^2^), mean and standard deviation	27.6 (SD 5.2)	28.7 (SD 5.4)	0.47
Type II diabetes	28.6 (6)	30.2 (13)	0.88
Diabetic nephropathy	28.6 (6)	20.9 (9)	0.54
Dyslipidemia treated with statins	57.1 (12)	62.8 (27)	0.87
Dialysis	90.5 (19)	90.7 (39)	0.98
Time elapsed between start of dialysis and colonoscopy and standard deviation	2.40 (SD 3.17)	1.90 (SD 2.98)	0.62
	95% CI (0.65 - 4.16)	95% CI (0.7 - 3.10)	
Immunosuppressive use	9.5 (2)	20.9 (9)	0.43

**Table 3:** Colonoscopies According to Patient’s Age

	Colonoscopies in < 50 years old (n = 19)	Colonoscopies in ≥ 50 years old (n = 45)
Screening colonoscopy % (n = 45)	57.9 (11)	75.6 (34)
% of patients with polyps (n = 21)	26.3 (5)	35.6 (16)
Adenomas % (n = 14)	15.8 (3)	24.4 (11)

There were no statistically significant differences between the groups.

**Table 4:** Endoscopic and Pathologic Results

	All patient with a colonoscopy % (n = 64)	Screening colonoscopies % (n = 45)	Diagnostic colonoscopies % (n = 19)	Patients with polyps % (n = 21)
% of patients with polyps	32.8 (21)	33.3 (15)	31.6 (6)	100 (21)
Polyps ≥ 10 mm	6.3 (4)	6.7 (3)	5.3 (1)	21.1 (4)
Adenomas	21.9 (14)	24.4 (11)	15.8 (3)	66.7 (14)
Hyperplastic polyps	4.7 (3)	4.4 (2)	5.3 (1)	9.5 (2)
Unknown pathology	6.3 (4)	4.4 (2)	10.5 (2)	19 (4)
Patients with multiple polyps (≥ 2)	20.3 (13)	20 (9)	21.1 (4)	61.9 (13)
Patients with only distal polyps	10.3 (7)	15.6 (7)	0	33.3 (7)
Patients with only proximal polyps	10.3 (7)	8.9 (4)	15.8 (3)	33.3 (7)
Patients with synchronous polyps	10.3 (7)	8.9 (4)	15.3 (3)	33.3 (7)
Diverticulosis	40.6 (26)	46.7 (21)	26.3 (5)	33.3 (7)
Angiodysplasia	4.7 (3)	4.4 (2)	5.3 (1)	4.8 (1)

## Results

### General population

A total of 179 patients received a kidney transplant during the study period. Twenty patients were excluded based on their re-transplant status. Of the 159 remaining patients, 50% (n = 79) had a test for colorectal screening (64 colonoscopy and 15 DCBE) in the 5 years preceding the transplantation. Sixty-seven point five percent of the patients over 50 years old at the time of the transplant had a pre-transplant colonoscopy as opposed to 14.6% of the patients less than 50 years old. Six other patients had a colonoscopy more than 5 years before the transplant (and therefore were not considered in our results). The clinical characteristics of our population and groups of patients who had a colonoscopy or who had polyps are shown in Table 1, 2.

### Colonoscopy

A colonoscopy was performed in the 5 years preceding the transplant in 64 patients. Pre-transplant screening was the indication for 70.3% of the colonoscopies (n = 45). The other indications were anemia (n = 5), acute (n = 5) and chronic (n = 3) lower gastrointestinal bleeding (LGIB), melena (n = 1), changes in bowel movements (n = 3) and as a diagnostic exam for lesion detected by another test (n = 2). The mean time elapsed between the start of dialysis and the colonoscopy was 2.09 years (SD 3.06; 95% CI 1.11 - 3.07). The mean time elapsed between colonoscopy and transplantation was 2.47 years (SD 1.28; 95% CI 2.15 - 2.78). Less than 10% of our population who had a colonoscopy had a family history of colorectal cancer, personal history of polyps, inflammatory bowel disease or other neoplasia. Types of cancer included skin cancers (basal cell carcinoma, melanoma *in situ*, squamous cell carcinoma), urologic cancers (clear cells renal cells carcinoma, papillary renal cells carcinoma, epithelioma of the bladder) and a squamous papilloma of the esophagus.

Total colonoscopies (up to the cecum) were done in 87.5% of patients with colonoscopies, in 95.2% of patients with polyps and in 83.7% of patients without polyps. The results according to patient’s age (< or ≥ 50 years old) are shown in Table 3. The overall polyp rate in patients less than 50 years old was 26.3%. However, only 11 out of 19 colonoscopies were for screening. In these 11 colonoscopies, four patients had polyps and three had adenomas. No proximal lesions were detected. Thus, screening colonoscopies in our population of less than 50 years old show an even higher rate of lesions than the general population less than 50 years old, with 36.4% of polyps and 27.3% of adenomas.

**Figure 1:**
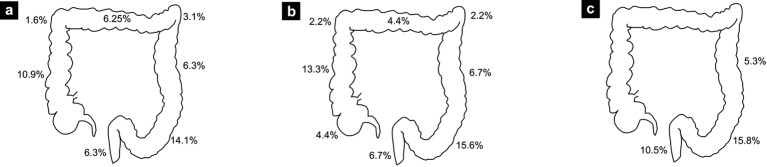
Percentage of patients with at least one lesion found at specific colon sites. (a) All colonoscopies. (b) Screening colonoscopies. (c) Patients under age of 50.

**Figure 2:**
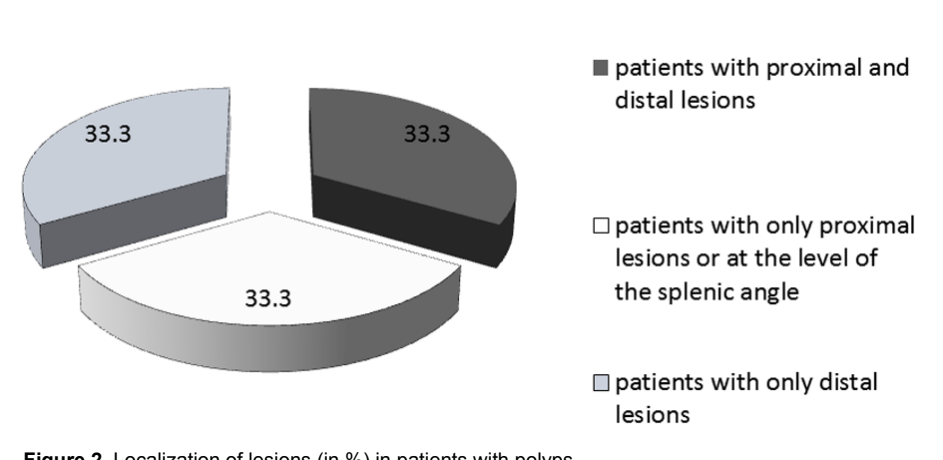
Localization of lesions (in %) in patients with polyps.

The endoscopic and pathologic results are shown in Table 4. Thirty-three percent of patients had polyps (n = 21). Among those patients, a total of 14 had proximal polyps, seven had synchronous and seven had only distal polyps. Four patients had advanced polyps (6.7%), thus a total of 10 polyps of 10 mm or greater and one tubulovillous adenoma. Three of these patients had the colonoscopy for screening and one patient because of LGIB. Figure 1 shows the proportion of patients according to the localization of their lesions and Figure 2 shows the proportion of proximal lesions.

No statistically significant differences were found between the groups with and without polyps in relation to their lifestyle, history of colorectal polyps, comorbidities, primary renal disease or immunosuppressive drug use. Also, no statistically significant difference was found between polyp prevalence in younger or older than 50 years old patients (Table 2), neither in groups with and without metabolic syndrome (32.4% vs. 33.3%; P = 0.33) or with and without diabetic nephropathy who had a colonoscopy (40.0% vs. 30.6%; P = 0.48).

No major adverse events occurred; only two patients had bleeding associated with polyp removal (3.13%), which was controlled by thermocoagulation (n = 1) and adrenaline injection (n = 1).

### DCBEs

Pre-transplant screening was the indication for all patients (n = 15) and all DCBEs were negative for lesions. Twelve patients were aged more than 50 years old at the time of the exam.

## Discussion

This study reports an overall prevalence of polyps and adenomas of 32.8% and 21.9% respectively in all patients with colonoscopy, and a prevalence of those in screening colonoscopies of 33.3% and 24.4%, respectively, which correspond to the literature for general population screening [23-26].

For subjects under the age of 50 years old, data from literature reveal a polyp prevalence of 18-21.1% and adenoma prevalence of 9.5-16.9% [19, 26, 27]. However, our screening-purpose colonoscopies in those patients showed a polyp prevalence of 36.4%, which was even higher than our rate for combined diagnostic and screening colonoscopy of 26.3%. Lee et al also reported a high lesion rate of 31% in the under 50 age group [19]. The small size of that subgroup and the ratio of screening-purpose colonoscopies (only 57% of colonoscopies were for screening) may explain the discordance between the rates of lesions among screening and overall colonoscopies. Also, some of these patients may have had an implicit indication for an earlier screening, as three patients had a possible family history of colorectal lesions. A prospective study targeting this population would be of interest to thoroughly evaluate whether these patients have a higher risk of developing earlier colonic lesions. This suggests, however, that a pre-transplant colonoscopy would also be of interest for patients under the age of 50, notably because of some data suggesting an association between post-transplant neoplasia and a younger age at transplant [28].

Our screening colonoscopy rate of 6.7% of advanced lesions agrees with data from the literature (5.0-9.6%) [22-26]. Moreover, only 8.9% of our patients had proximal lesions and another 8.9% had synchronous lesions (thus an overall proximal lesions rate of 17.8%). These data are in agreement with the literature (4-30.5% with at least one proximal lesion and 6-14.6% with only proximal lesions [21-26]). The rate in patients 50 and older is even higher, with 26.6% of patients having at least one proximal lesion. Lee et al mentioned a polyp rate at the level of the ascending colon of 50% compared to 33.3% of our patients with polyps [19]. In this group (all colonoscopy indications included), 66.7% had proximal lesions and 33.3% had only lesions proximal to or at the level of the splenic angle. Several studies also mention a higher rate of proximal lesions in particular groups, notably individuals with chronic kidney disease [12, 19], women [29], Afro-Americans [30], patients over the age of 60 years old [26, 31], post-cholecystectomy [32] and those with characteristics of metabolic syndrome [13, 33]. The carcinogenesis mechanisms of proximal lesions may be different from those related to distal lesion, in part by an abnormal methylation of DNA causing microsatellite instability and a default in DNA repair mechanisms [31, 34]. Thus, our overall proximal lesion rate of 10.3% and the possible increased risk of proximal lesions among kidney transplant candidates provide a compelling argument for the more appropriate use of total colonoscopies in this population.

ESRD has intrinsic neoplasia risk factors, including viral infections (Epstein-Barr virus, human papilloma virus, hepatitis B and C viruses), uremia (which leads to an immunocompromised state) [35], some nutritional deficiencies [36], retention of procarcinogens [37], DNA hypomethylation, defects in DNA repair mechanisms [18] and complement activation by dialysis membrane [18]. The length of the ESRD state and the duration of dialysis before colonoscopy could prove to be interesting risk factors [38]. Time elapsed before neoplasia diagnosis varies (for any kind of neoplasia), with a mean of 2.8 to 6.37 years [2, 4, 5]; the majority of neoplasia cases reported by Cengiz et al had a diagnosis of chronic kidney disease for less than 10 years [2]. However, in our study a mean of only 2.09 years elapsed between the initiation of dialysis and the colonoscopy, which could be insufficient to significantly increase the risk of neoplasia.

The time elapsed before an adenoma becomes a carcinoma depends on its size, histology and the patient age, varying between 3.6 and 9.5 years [39]. After 10 years, 25.2-43.3% of advanced adenomas would become carcinomas [40]. The increased prevalence of colorectal neoplasia in renal transplant recipients is the subject of several studies [4, 17, 20-22, 28, 41]. A case-control Korean study showed a significantly increased risk of any colorectal lesions among renal transplant recipients. The duration of immunosuppression therapy would be an important factor [17] and some studies have shown a steady increase in the risk of post-transplant neoplasia according to the time elapsed after the transplant [28, 41].

This real-life observational study reports the results of the colonoscopies done before kidney transplantation at our center. Since the CHUM is a referral center for transplantation in Quebec, we had very extensive data from the pre-transplant workup of candidates. The major limitation of this study is its retrospective and observational character. Many patients of our cohort did not have any screening exam (n = 80), but 84% of them were aged less than 50 years old at the time of the transplant and screening for colorectal cancer is not recommended in most of this population [42]. Moreover, among patients aged 50 and older, almost 75% of our cohort had a screening exam (colonoscopy or DCBE), which reinforces the validity of our results in this population.

However, of our 64 colonoscopies, 30% were performed as a result of signs and symptoms, thus perhaps overestimating the prevalence of lesions in renal transplant candidates (case spectrum bias). However, our results may also underestimate the prevalence and severity of pre-transplantation lesions. First, pathology was unknown for less than 20% of our patients with lesions, sometimes because the referring center did not send the pathology report and sometimes because the polyp was lost at removal. Secondly, due to the retrospective character of our study, some patients with worrisome pre-transplantation findings could have been excluded from the transplant list, and thus, perhaps, were not in our cohort. This study shows the prevalence of pre-transplant lesions among our renal transplant recipients. It is therefore probably not representative of colorectal findings in overall kidney transplant candidates.

Nonetheless, the pre-transplantation polyp rate is high in our renal transplant recipients, pointing to the necessity of pre- and post-transplant colorectal cancer screening. Moreover, the high rate of proximal lesions suggests the need to perform a total colonoscopy for screening. The increase of post-transplantation colorectal neoplasia is the subject of several studies [4, 17, 20-22, 28, 41], and systematic follow-up could be recommended, considering the high pre-transplantation prevalence of lesions.

## References

[1] National Kidney and Urologic Diseases Information Clearinghouse. Kidney Disease Statistics for the United States U.S.. Department of Health and Human Services National Institutes of Health. NIH Publication. No. 12-3895. July, 2012.

[2] Cengiz K (2002). Increased incidence of neoplasia in chronic renal failure (20-year experience). Int Urol Nephrol.

[3] Wong G, Hayen A, Chapman JR, Webster AC, Wang JJ, Mitchell P, Craig JC (2009). Association of CKD and cancer risk in older people. J Am Soc Nephrol.

[4] Vajdic CM, McDonald SP, McCredie MR, van Leeuwen MT, Stewart JH, Law M, Chapman JR (2006). Cancer incidence before and after kidney transplantation. JAMA.

[5] Maisonneuve P, Agodoa L, Gellert R, Stewart JH, Buccianti G, Lowenfels AB, Wolfe RA (1999). Cancer in patients on dialysis for end-stage renal disease: an international collaborative study. Lancet.

[6] U.S. Cancer Statistics Working Group (2012). United States Cancer Statistics: 1999-2008 Incidence and Mortality Web-based Report. Atlanta: U.S. Department of Health and Human Services, Centers for Disease Control and Prevention and National Cancer Institute.

[7] Iseki K, Osawa A, Fukiyama K (1993). Evidence for increased cancer deaths in chronic dialysis patients. Am J Kidney Dis.

[8] Inamoto H, Ozaki R, Matsuzaki T, Wakui M, Saruta T, Osawa A (1991). Incidence and mortality patterns of malignancy and factors affecting the risk of malignancy in dialysis patients. Nephron.

[9] Ito T, Tanaka I, Kadoya T, Kimura M, Ooshiro T, Ooishi K, Tanaka J (1999). Screening for gastroenterological malignancies in new and maintenance dialysis patients. J Gastroenterol.

[10] Kasiske BL, Ramos EL, Gaston RS, Bia MJ, Danovitch GM, Bowen PA, Lundin PA (1995). The evaluation of renal transplant candidates: clinical practice guidelines. The evaluation of renal transplant candidates: clinical practice guidelines. J Am Soc Nephrol.

[11] Knoll G, Cockfield S, Blydt-Hansen T, Baran D, Kiberd B, Landsberg D, Rush D (2005). Canadian Society of Transplantation: consensus guidelines on eligibility for kidney transplantation. CMAJ.

[12] Bini EJ, Kinkhabwala A, Goldfarb DS (2006). Predictive value of a positive fecal occult blood test increases as the severity of CKD worsens. Am J Kidney Dis.

[13] Kim JH, Lim YJ, Kim YH, Sung IK, Shim SG, Oh SO, Park SS (2007). Is metabolic syndrome a risk factor for colorectal adenoma?. Cancer Epidemiol Biomarkers Prev.

[14] Giovannucci E, Ascherio A, Rimm EB, Colditz GA, Stampfer MJ, Willett WC (1995). Physical activity, obesity, and risk for colon cancer and adenoma in men. Ann Intern Med.

[15] Poynter JN, Gruber SB, Higgins PD, Almog R, Bonner JD, Rennert HS, Low M (2005). Statins and the risk of colorectal cancer. N Engl J Med.

[16] Rostami Z, Einollahi B, Lessan-Pezeshki M, Nourbala MH, Nemati E, Pourfarziani V, Shahbazian H (2011). Old male living renal transplant recipients more likely to be at risk for colorectal cancer. Transplant Proc.

[17] Park JM, Choi MG, Kim SW, Chung IS, Yang CW, Kim YS, Jung CK (2010). Increased incidence of colorectal malignancies in renal transplant recipients: a case control study. Am J Transplant.

[18] Vamvakas S, Bahner U, Heidland A (1998). Cancer in end-stage renal disease: potential factors involved -editorial. Am J Nephrol.

[19] Lee S, Wasserberg N, Petrone P, Rosca J, Selby R, Ortega A, Kaufman HS (2008). The prevalence of colorectal neoplasia in patients with end-stage renal disease: a case-control study. Int J Colorectal Dis.

[20] Collins MG, Teo E, Cole SR, Chan CY, McDonald SP, Russ GR, Young GP (2012). Screening for colorectal cancer and advanced colorectal neoplasia in kidney transplant recipients: cross sectional prevalence and diagnostic accuracy study of faecal immunochemical testing for haemoglobin and colonoscopy. BMJ.

[21] Kasiske BL, Snyder JJ, Gilbertson DT, Wang C (2004). Cancer after kidney transplantation in the United States. Am J Transplant.

[22] Webster AC, Craig JC, Simpson JM, Jones MP, Chapman JR (2007). Identifying high risk groups and quantifying absolute risk of cancer after kidney transplantation: a cohort study of 15,183 recipients. Am J Transplant.

[23] Ferlitsch M, Reinhart K, Pramhas S, Wiener C, Gal O, Bannert C, Hassler M (2011). Sex-specific prevalence of adenomas, advanced adenomas, and colorectal cancer in individuals undergoing screening colonoscopy. JAMA.

[24] Schoenfeld P, Cash B, Flood A, Dobhan R, Eastone J, Coyle W, Kikendall JW (2005). Colonoscopic screening of average-risk women for colorectal neoplasia. N Engl J Med.

[25] Strul H, Kariv R, Leshno M, Halak A, Jakubowicz M, Santo M, Umansky M (2006). The prevalence rate and anatomic location of colorectal adenoma and cancer detected by colonoscopy in average-risk individuals aged 40-80 years. Am J Gastroenterol.

[26] Lieberman DA, Weiss DG, Bond JH, Ahnen DJ, Garewal H, Chejfec G (2000). Use of colonoscopy to screen asymptomatic adults for colorectal cancer. Use of colonoscopy to screen asymptomatic adults for colorectal cancer. N Engl J Med.

[27] Regula J, Rupinski M, Kraszewska E, Polkowski M, Pachlewski J, Orlowska J, Nowacki MP (2006). Colonoscopy in colorectal-cancer screening for detection of advanced neoplasia. N Engl J Med.

[28] Adami J, Gabel H, Lindelof B, Ekstrom K, Rydh B, Glimelius B, Ekbom A (2003). Cancer risk following organ transplantation: a nationwide cohort study in Sweden. Br J Cancer.

[29] Li FY, Lai MD (2009). Colorectal cancer, one entity or three. J Zhejiang Univ Sci B.

[30] Thornton JG, Morris AM, Thornton JD, Flowers CR, McCashland TM (2007). Racial variation in colorectal polyp and tumor location. J Natl Med Assoc.

[31] Lindblom A (2001). Different mechanisms in the tumorigenesis of proximal and distal colon cancers. Curr Opin Oncol.

[32] Reid FD, Mercer PM, harrison M, Bates T (1996). Cholecystectomy as a risk factor for colorectal cancer: a meta-analysis. Scand J Gastroenterol.

[33] Giovannucci E (2007). Metabolic syndrome, hyperinsulinemia, and colon cancer: a review. Am J Clin Nutr.

[34] Imperiale TF, Wagner DR, Lin CY, Larkin GN, Rogge JD, Ransohoff DF (2002). Results of screening colonoscopy among persons 40 to 49 years of age. N Engl J Med.

[35] Schollmeyer P, Bozkurt F (1988). The immune status of the uremic patient: hemodialysis vs CAPD. Clin Nephrol.

[36] Kallistratos G, Evangelou A, Seferiadis K, Vezyraki P, Barboutis K (1985). Selenium and haemodialysis: serum selenium levels in healthy persons, non-cancer and cancer patients with chronic renal failure. Nephron.

[37] Yanagisawa H, Wada O (1989). Significant increase of IQ-type heterocyclic amines, dietary carcinogens in the plasma of patients with uremia just before induction of hemodialysis treatment. Nephron.

[38] Wong G, Turner RM, Chapman JR, Howell M, Lim WH, Webster AC, Craig JC (2013). Time on dialysis and cancer risk after kidney transplantation. Transplantation.

[39] Chen CD, Yen MF, Wang WM, Wong JM, Chen TH (2003). A case-cohort study for the disease natural history of adenoma-carcinoma and de novo carcinoma and surveillance of colon and rectum after polypectomy: implication for efficacy of colonoscopy. Br J Cancer.

[40] Brenner H, Hoffmeister M, Stegmaier C, Brenner G, Altenhofen L, Haug U (2007). Risk of progression of advanced adenomas to colorectal cancer by age and sex: estimates based on 840,149 screening colonoscopies. Gut.

[41] Kyllonen L, Salmela K, Pukkala E (2000). Cancer incidence in a kidney-transplanted population. Transpl Int.

[42] Zauber AG, Lansdorp-Vogelaar I, Knudsen AB, Wilschut J, van Ballegooijen M, Kuntz KM (2009). Evaluating Test Strategies for Colorectal Cancer Screening-Age to Begin, Age to Stop, and Timing of Screening Intervals: A Decision Analysis of Colorectal Cancer Screening for the U.S. Preventive Services Task Force from the Cancer Intervention and Surveillance Modeling Network (CISNET). Rockville (MD).

